# Autophagy in Gastric Mucosa: The Dual Role and Potential Therapeutic Target

**DOI:** 10.1155/2021/2648065

**Published:** 2021-06-11

**Authors:** Sheng-Yu Lu, Song Guo, Shao-Bin Chai, Jia-Qi Yang, Yuan Yue, Hao Li, Pei-Ming Sun, Tao Zhang, Hong-Wei Sun, Jin-Lian Zhou, Jian-Wu Yang, He-Ming Yang, Zheng-Peng Li, Yan Cui

**Affiliations:** ^1^Department of General Surgery, The 306th Hospital of PLA-Peking University Teaching Hospital, Beijing 100101, China; ^2^Department of General Surgery, Strategic Support Force Medical Center, Beijing 100101, China; ^3^Department of Pathology, Strategic Support Force Medical Center, Beijing 100101, China; ^4^Department of Gastroenterology and Hepatology, The First Medical Center, Chinese PLA General Hospital, Beijing 100853, China

## Abstract

The incidence of stomach diseases is very high, which has a significant impact on human health. Damaged gastric mucosa is more vulnerable to injury, leading to bleeding and perforation, which eventually aggravates the primary disease. Therefore, the protection of gastric mucosa is crucial. However, existing drugs that protect gastric mucosa can cause nonnegligible side effects, such as hepatic inflammation, nephritis, hypoacidity, impotence, osteoporotic bone fracture, and hypergastrinemia. Autophagy, as a major intracellular lysosome-dependent degradation process, plays a key role in maintaining intracellular homeostasis and resisting environmental pressure, which may be a potential therapeutic target for protecting gastric mucosa. Recent studies have demonstrated that autophagy played a dual role when gastric mucosa exposed to biological and chemical factors. More indepth studies are needed on the protective effect of autophagy in gastric mucosa. In this review, we focus on the mechanisms and the dual role of various biological and chemical factors regulating autophagy, such as *Helicobacter pylori*, virus, and nonsteroidal anti-inflammatory drugs. And we summarize the pathophysiological properties and pharmacological strategies for the protection of gastric mucosa through autophagy.

## 1. Introduction

Gastric diseases mainly include chronic gastritis, peptic ulcer, and gastric cancer. Gastric diseases are very common with a high morbidity, and its impact on human health is a worldwide problem in modern society [1]. Damaged gastric mucosa is more vulnerable to injury, leading to bleeding and perforation, which eventually aggravates the primary disease [2]. Gastric mucosal protection is also needed during severe trauma, shock, infection, burns, and surgery to avoid the formation of gastric ulcers [3]. The protection of gastric mucosa is always a major clinical challenge, and many indepth researches have been conducted to explore the mechanism of gastric mucosal protection. Various drugs are used to treat gastric mucosa damage, including antacids, proton-pump inhibitors, and histamine H_2_-receptor antagonists. However, these drugs could cause nonnegligible side effects, such as hepatic inflammation, nephritis, hypoacidity, impotence, osteoporotic bone fracture, and hypergastrinemia [4, 5]. Accordingly, many studies have focused on investigating novel approaches for the prevention of gastric mucosal damage.

Autophagy has been proved to play a vital role in the regulation of various cellular functions. Autophagy is a major cellular protective mechanism, which can help cells adapt to the changing environment, protect cells from harmful factors, and finally maintain the dynamic balance of cells [6]. Recent studies have shown that autophagy may be a double-edged sword, and various factors can destroy and protect gastric mucosa through autophagy. Therefore, the mechanism of autophagy in gastric mucosa needs to be clarified. We reviewed the current literatures to discuss the role of autophagy in gastric mucosal cell biology and to summarize pathophysiological properties and pharmacological strategies for the protection of gastric mucosa.

## 2. Gastric Mucosal Barrier

The gastric mucosal barrier consists of preepithelial layer, dense epithelial components, postepithelial layer, microcirculation, and nerves. Epithelial cells are responsible for the integrity and function of the gastric mucosal barrier [7]. The gastric mucosal barrier is a complex system involving physical, chemical, and biological defense mechanisms that protect the stomach from irritating food, gastric acid, and excessive pepsin activity [8].

Gastric mucosa develops many defense mechanisms against adverse gastric environment, including mucus-bicarbonate-phospholipid barrier, mucous layer, mucosal microcirculation, epithelial cell renewal, endogenous prostaglandins, and epidermal growth factor. The environment in the stomach is always in sharp fluctuations. Exogenous factors such as stress, infection, excessive intake of alcohol, or long-term drug consumption can cause injury which leads to gastric diseases [9, 10]. Gastric contents such as gastric acid, protein hydrolysate, duodenal bile, and ethanol have a great impact on stomach itself [11–13]. Stomach repair and damage are in a delicate balance. The destruction of gastric mucosal integrity is a result of an imbalance between defensive factors and the aggressive factors [14].

The disruption of balance in stomach can lead to mild or serious consequences. Gastritis and gastric ulcers are mild cases while some of severe cases will gradually worsen, leading to unexpected complications, such as bleeding or perforation [15]. The maintenance of the gastric barrier is very important, in which the maintenance of gastric mucosal epithelium is the key aspect.

## 3. Autophagy

Autophagy is a general term for the transmission of cytoplasmic substances to lysosomes in animal cells or vacuoles in plant and yeast cells [16]. Autophagy can be divided into three types: macroautophagy, microautophagy, and chaperone-mediated autophagy [17].

Autophagy plays a key role in homeostasis. The target substance of autophagy is recycled to create new cellular structures or as a source of energy [18]. Autophagy is not just a simple metabolic process. Studies have clearly shown that autophagy has richer physiological and pathophysiological effects than expected, such as starvation adaptation, intracellular protein and organelle clearance, development, antiaging, microbial elimination, cell death, tumor inhibition, and antigen presentation [19].

Autophagy mainly refers to macroautophagy. Macroautophagy is a metabolic process in which cells wrap proteins or organelles to form autophagosomes through bilayer membranes. Then, the outer membrane fuses with the lysosomal membrane to form autolysosomes and finally degrades the wrapped contents by hydrolyzing enzymes [20]. The process of autophagy begins with the formation of autophagosomes, which includes initiation, nucleation, prolongation, and closure. ULK-51-like kinase (ULK) complex (ULK1-Atg13-Atg17) and the class III phosphatidylinositol 3-kinase (PtdIns3K) complexes regulate early steps in the autophagosome formation. Two ubiquitin-like proteins, including Atg12-Atg5-Atg16 and microtubule-associated protein 1 light chain 3 (LC3) conjugation systems, mediate vesicle elongation and expansion. Then, the autophagosome fuses with the lysosome to form autolysosome. Lysosomal membrane protein LAMP2 and the small GTPase RAB7 are implicated in autophagosome-lysosome fusion and degradation of the cargo by a series of hydrolases [21].

### 3.1. Biological and Chemical Factors Modulating Autophagy

As a part of the digestive system, the stomach is hit by various biological and chemical factors, such as bacteria, viruses, biotoxins, ethanol, and drugs. The most clinically significant factor is *Helicobacter pylori*, we discuss it as a separate section, and the others are included in one section (Table 1).

### 3.2. H. pylori

*H. pylori*, a Gram-negative bacterium that colonizes the stomach, is accepted as one of the leading causes of several gastroduodenal diseases, including gastritis, peptic ulcer, and gastric cancer, and it has been classified as a human carcinogen while the eradication of *H. pylori* can prevent the occurrence of gastric cancer [46, 47].

In acute *H. pylori* infection, various factors cooperate and antagonize each other. *H. pylori* produces numbers of virulence factors that are involved in pathogenesis of disease, such as urease, flagellum, vacuolating cytotoxin (VacA), and protein of cytotoxin-associated gene A (CagA) [48]. Among them, VacA and CagA are extremely crucial [49]. Available evidence indicated that *H. pylori* infection induced autophagy in gastric epithelial cells in a VacA-dependent manner. VacA was necessary for inducing autophagy in cultured AGS cells and was sufficient to induce autophagy alone. The regulation of autophagy by VacA seems to be two-sided. VacA-induced autophagy served as a host mechanism to limit toxin-induced cellular damage [22]. On the contrary, VacA-induced mitochondrial damage leading to apoptosis was believed to be a major cause of cell death, and low-density lipoprotein receptor-related protein-1 (LRP1) was one of the receptors [23]. Similarly, Zhu and colleagues found that VacA also induced autophagic cell death via the endoplasmic reticulum stress in AGS cells [24]. A recent study showed that connexin was involved in VacA-induced cell death. *H. pylori* VacA induced apoptosis by accumulating connexin 43 in autophagy vesicles through the rac1/ERK-dependent pathway [25]. Another virulence factor, CagA, is closely related to VacA. Studies have shown that CagA was degraded through autophagy and therefore had a short lifetime [50]. At the same time, VacA-mediated autophagy destruction promoted the accumulation of CagA in cells which could accelerate the development of gastric cancer [26]. There was an evidence that VacA might not be the only factor associated with *H. pylori*-mediated autophagy in epithelial cells. CagA protein, negatively regulating autophagy and facilitating the expression of proinflammatory cytokines (IL-8, TNF-*α* and IL-1b), promoted inflammation through the c-Met-PI3K/AKT-mTOR signaling pathway [27]. Similarly, *H. pylori* secreting *HP0175* induced autophagy which was independent of VacA, and its mechanism was related to unfolded protein response (UPR) [28]. *H. pylori* inhibited p14ARF tumor suppressor gene and promoted the occurrence of cancer by inducing p14ARF degradation. Meanwhile, p14ARF regulated autophagy, and the degradation of p14ARF led to the decrease of autophagy [29].

In acute infection, the destruction of lysosome function should be noted. Autophagy induced by microbes involving the trafficking of intracellular bacteria in the lysosomes is known as xenophagy. In recent years, xenophagy has increasingly been recognized as a key mechanism for defending against bacteria [51]. As a natural defense mechanism, autophagy can kill intracellular microorganisms [52]. However, several successful intracellular pathogens can also improve their own survival through autophagy [53]. *H. pylori* is generally recognized as a noninvasive bacterium adhering to the gastric epithelium surface, whereas several in vitro studies suggested the occurrence of bacteria invading into gastric epithelial cells [54]. Current evidence suggests that *H. pylori* can invade epithelial cells, macrophages, and dendritic cells [28]. The ability of *H. pylori* to reside in gastric epithelial cells may be part of the reason why it is difficult to eradicate infection with antimicrobial therapy. Short-term exposure to VacA affected the gastric mucosal function by increasing autophagy, and at the same time, the function of lysosome was also impaired [30]. VacA could destroy the activity of transient receptor potential membrane channel mucolipin 1 (TRPML1), which inhibits lysosome and autophagy killing and promotes bacterial colonization [31]. Similarly, capping actin protein of muscle Z-line alpha subunit 1 (CAPZA1) negatively regulated the formation of autolysomes by inhibiting the expression of lysosomal-associated membrane protein 1 (LAMP1), thus inhibiting CagA-degraded autophagy and promoting the development of gastric cancer [32]. Zhang and his colleagues also found that *H. pylori* destroyed the degradation function of autolysome and made it isolated in the nondegradative autophagosomes of gastric epithelial cells, thus enhancing its colonization ability [33].

Autophagy in gastric mucosal cells decreases in chronic persistent infection. MicroRNA forms a complex regulatory network and has a vital role in the control of inflammatory response associated with *H. pylori* infection [55]. *H. pylori* upregulated Mir-30b in persistent infected gastric mucosal cells and inhibited autophagy by targeting ATG12 and BECN1 [35]. Mir-30d downregulated the expression of key autophagy genes ATG2B, ATG5, ATG12, BECN1, and BNIP3L and inhibited the autophagy response of gastric epithelial cells to *H. pylori* invasion, thus increasing the intracellular survival rate of *H. pylori* [36]. On the contrary, Mir-155 enhanced autophagy in human gastric epithelial cells and decreased the survival of intracellular *H. pylori* [37]. The signal transducer and activator of transcription 3 (STAT3) is an important transcription factor of the JAK/STAT signal pathway, mediating the expression of various genes [56]. *H. pylori* infection induced phosphorylation of STAT3 on Ser727, which led to increased autophagy and mitochondrial damage [38]. Since *H. pylori* cannot be cocultured with cells for a long time, *H. pylori* lysates were used insteading of living bacteria to coculture with gastric epithelial cells for a long time to simulate the regulatory effects of persistent infection on cells. GES-1 was cocultured with *H. pylori* lysates for 30 generations. *H. pylori* lysates inhibited apoptosis and autophagy through the Nod1-NF-*κ*B/MAPKERK/FOXO4 signaling pathway, which was conducive to the continuous colonization of *H. pylori*, thus promoting the development of gastric cancer [39]. After long exposure to VacA, the lack of cathepsin D in autophagosomes of gastric mucosal cells blocked the induction of autophagy and led to the decrease of autophagy [34].

### 3.3. Other Factors Inducing Autophagy

In addition to the indepth study of the *H. pylori* effect on autophagy of gastric mucosal cells, some other factors often exposed to the environment have also been shown to affect gastric mucosal autophagy. Autophagy can be activated in a series of damage responses, including excessive ROS (reactive oxygen species), caused by oxidative stress to eliminate toxic polymers and damaged organelles [57]. Ethanol-induced gastric mucosal injury may be the result of oxidative stress caused by excessive production of ROS. At the same time, ethanol activated autophagy by downregulating the mTOR signal pathway. Autophagy plays a protective role in the injury of gastric mucosa induced by ethanol, which inhibits the production of ROS and the degradation of antioxidants and lipid peroxidation [40]. The damaging effect of ROS can also be seen in biotoxins. Ochratoxin A (OTA), one of the most important and deleterious mycotoxins worldwide, is found ubiquitously in numerous foodstuffs and feedstuffs. OTA increased the autophagy flux of gastric mucosal cells through the AMPK/mTOR pathway and led to autophagic cell death [41]. Autophagy also seems to be related to the regulation of inflammation. EV71 not only induced autophagy in GES-1 cells but also promoted the expression and release of IL-6, which was regulated by the P38MAPK/ERK pathway [42]. Nonsteroidal anti-inflammatory drugs (NSAIDs) are a classic drug that damages gastric mucosa. Recent study found that sulindac sulfide downregulated autophagic death induced by survivin as a new injury mechanism [43]. Similarly, other study showed that aspirin-induced gastrointestinal injury was related to the inhibition of epithelial cells autophagy. Aspirin inhibited autophagy of rat gastric mucosa and human gastric epithelial cells through mTOR-mediated ULK1 phosphorylation. Further inhibition of autophagy aggravated aspirin-induced gastric injury and epithelial cell apoptosis [44]. Euphorbia factor L1 (EFL1) reduced the survival rate of GES-1 cells by inhibiting the PI3K/AKT/mTOR signal pathway, inducing oxidative stress and activating mitochondrial-mediated apoptosis and autophagy [45].

### 3.4. Drugs Protecting Gastric Mucosa through Autophagy

Autophagy is strictly controlled by different regulatory signals. Since autophagy is a promising therapeutic target, a lot of works have been focused on extracellular stimulators that can enhance or inhibit autophagy to protect gastric mucosa. At present, more and more evidences show that various chemical factors (clinical drugs, cytokines, and natural compounds) can affect the autophagy ability of gastric mucosal cells and then protect gastric mucosa. We conclude some valuable chemical factors protecting gastric mucosa by affecting autophagy, which may become a new target for gastric mucosal protection and treatment of gastric ulcer (Table 2).

*H. pylori* eradication is recognized to be the fundamental means for the treatment of gastric ulcers [70]. Nowadays, the combination of proton-pump inhibitors and antibiotics is the first choice for the treatment of *H. pylori* infection, but the increased resistance to antibiotics weakens the effectiveness of antibiotics [71–73]. Autophagy seems to be a new target for the treatment of *H. pylori*. The treatment of catechin combined with sialic acid inhibited apoptosis and enhanced autophagy, thus making gastric mucosal cells resist*H. pylori* infection [58, 59]. The mechanism was that it promoted autophagy, thus reducing the activation of caspase-1 [60]. The expression of IFN-*γ* was upregulated in the stomach of humans and mice infected with *H. pylori*. IFN-*γ* inhibited epithelial cell apoptosis, normalized cell proliferation, and promoted Beclin1-mediated autophagy [61]. The function of lysosome was destroyed by *H. pylori* infection, making autophagy lysosome become the replication site of bacteria. Vitamin D3 restored the degradation function of lysosome by activating the PDIA3-STAT3-MCOLN3-Ca2+ axis, which led to the increase of lysosomal calcium release and the normalization of lysosomal acidification. The recovery of lysosome degradation function promoted the clearance of *H. pylori* through the autolysome pathway [62]. Astaxanthin (AST) is a powerful antioxidant. Astaxanthin activated AMPK and inhibited its downstream target molecule mTOR to induce autophagy. Through this molecular mechanism, AST significantly inhibited *H. pylori*-induced apoptosis [63].

Pyrrolidine dithiocarbamate (PDTC) is an antioxidant. Intragastric administration of H_2_O_2_ can cause gastric injury and reduce gastric autophagy. However, PDTC treatment restored gastric injury and reduced H_2_O_2_-induced autophagy [64]. Acid and oxidative injury induce apoptosis while vitamin D3 combined to alginates activated autophagic and survival pathways to protect the gastric mucosa [65].

NSAIDs are classic drugs to protect gastric mucosa, and the mechanism of their inhibition of COX enzyme has been widely known [74]. Recent studies have also found that gastric mucosa is affected through autophagy. Chloroquine (CQ) is an ancient antimalarial drug and a very promising autophagy inhibitor for clinical application. It has the possibility of direct anticancer or chemotherapy enhancement [75]. The pharmacological effect of CQ on autophagy significantly reduced indomethacin-related gastric epithelial cytotoxicity [66]. CQ improved the mRNA and protein levels of Smad7, inhibited autophagy, and reduced indomethacin-induced apoptosis and autophagic death [67].

Polysaccharides from *Dendrobium officinale* Kimura and Migo Leaves (LDOP-1) attenuated ethanol-induced mucosal injury, significantly reduced the score of gastric mucosal injury and the degree of pathological injury and improved the ability of antioxidation. The mechanism was that the AMPK/mTOR signal pathway had a protective effect on ethanol-induced gastric mucosal injury [68].

The change of autophagy can also affect the occurrence of tumor [76]. Astragaloside IV (*AS-IV*) regulated the expression of p53, activated the Ambra1/Beclin1 complex in gastric precancerous lesions (GPL), decreased gastric mucosal injury, and prevented gastric mucosal atrophy, intestinal metaplasia, and atypical hyperplasia. It provided a potential therapeutic strategy for reversing intestinal metaplasia and dysplasia of gastric precancerous lesions and protecting gastric mucosa [69].

## 4. Conclusion

For a long time, the protection of gastric mucosa has been an important topic in clinic. Various drugs are used to treat gastric mucosal damage, including antacids, proton-pump inhibitors, and histamine H2-receptor antagonists and achieve good results. However, these medicines may cause nonnegligible side effects, such as hepatic inflammation, nephritis, hypoacidity, impotence, osteoporotic bone fracture, and hypergastrinemia. As a key process of cell adaptation to stress, autophagy plays an important role in the regulation of gastric mucosal cells and is expected to become a new target for gastric mucosal protection. The evidence reported in this paper is of great significance for understanding the role of autophagy in the biological function of gastric mucosal cells. A lot of work is devoted to discovering the mechanism and corresponding protective measures of biological and chemical factors such as *H. pylori* and NSAIDs affecting gastric mucosa through autophagy, but the exact mechanism behind this effect is still contradictory and ambiguous. The exact relationship between autophagy and its effect on the biological function of gastric mucosal cells needs to be further clarified. Future research should focus on the study of biological and chemical factors on the mechanism of autophagy in order to find new targets and develop drugs to protect gastric mucosa through autophagy.

## Figures and Tables

**Table 1:** Biological and chemical factors inducing autophagy in gastric mucosa.

Factors	Agent	Effect	Mechanism	Reference
*H. pylori* (acute infection)	VacA	Reduces the stability of VacA, and limits toxin-induced cellular damage	Promotes autophagy in a VacA-dependent manner	[22]
	VacA	Induces apoptosis	Promotes autophagy by VacA receptor LRP1	[23]
	VacA	Induces apoptosis	Induces autophagic cell death via the endoplasmic reticulum stress pathway	[24]
	VacA	Induces apoptosis	Accumulates connexin 43 in autophagy vesicles via a Rac1/ERK dependent pathway	[25]
	CagA	Promotes the development of gastric cancer	Promotes the accumulation of CagA through VacA-mediated autophagy destruction	[26]
	CagA	Promotes inflammation	Decreases autophagy via c-met-PI3K/Akt-mTOR signaling pathway	[27]
	HP0175	Induces apoptosis	Promotes autophagy by HP0175, link UPR	[28]
	—	Promotes the development of gastric cancer.	Induces degradation of p14ARF and downregulates autophagy	[29]
	VacA	Induces lysosomal damage	Promotes autophagy by galectin-8 aggregation	[30]
	VacA	Promotes the survival and colonization of *H. pylori*	Destroys the activity of TRPML1 and inhibits lysosome and autophagy killing	[31]
	CagA	Promotes the development of gastric cancer	Inhibits CagA-degraded autophagy and CAPZA1 negatively regulates the formation of autolysomes by inhibiting the expression of LAMP1	[32]
	—	Promotes the survival and colonization of *H. pylori*	Inhibits lysosomal clearance of autophagosomes	[33]

*H. pylori* (chronic infection)	VacA (prolonged exposure)	Promotes inflammation and the development of gastric cancer	Decreases autophagy and accumulates defective autophagosomes due to lack of cathepsin D	[34]
Mir-30b		Promotes the survival and colonization of *H. pylori*	Inhibits autophagy by targeting ATG12 and BECN1	[35]
Mir-30d		Promotes the survival and colonization of *H. pylori*	Inhibits autophagy pathway	[36]
Mir-155		Decreases the survival of intracellular *H. pylori*	Promotes autophagy	[37]
	—	Induces inflammation	Promotes autophagy by upregulating STAT3 phosphorylation on Ser727	[38]
*H. pylori* lysate		Promotes the survival and colonization of *H. pylori* and the development of gastric cancer	Inhibits apoptosis and autophagy through the Nod1-NF-*κ*B/MAPKERK/FOXO4 signaling pathway.	[39]
Ethanol		Induces gastric mucosal injury and active autophagy	Promotes autophagy by downregulating the mTOR signal pathway	[40]
Ochratoxin A		Induces apoptosis	Promotes autophagy by the AMPK/mTOR pathway	[41]
EV71		Induces inflammation	Increases autophagy, promotes the expression, and releases IL-6 by the P38MAPK/ERK pathway.	[42]
Sulindac sulfide		Induces apoptosis	Induces autophagic death via survivin downregulation	[43]
Aspirin		Induces gastrointestinal damage and apoptosis	Inhibits autophagy by activating mTOR-mediated ULK1 phosphorylation	[44]
EFL1		Induces apoptosis	Promotes autophagy by the PI3K/AKT/mTOR pathway	[45]

**Table 2:** Drugs protecting gastric mucosa by autophagy in gastric mucosa.

Chemical factor	Antagonist	Effects	Mechanism	Reference
Combination of catechins and sialic acid	*H. Pylori*	Protects AGS cells against *H. pylori* infection	Downregulates apoptosis and upregulates autophagy that reducing the activation of caspase-1	[58–60]
IFN-*γ*	*H. Pylori*	Inhibits gastric carcinogenesis	Induces epithelial cell autophagy	[61]
Vitamin D3	*H. Pylori*	Eliminates *H. pylori*	Restores lysosomal degradation function by activating the PDIA3-STAT3-MCOLN3-Ca2+ axis	[62]
Astaxanthin	*H. Pylori*	Inhibits gastric diseases associated with *H. pylori* infection	Increases autophagy through the activation of the AMPK pathway	[63]
PDTC	Oxidative stress induces by hydrogen peroxide (H_2_O_2_)	Restores gastric damages	Restores diminished autophagy induced by H_2_O_2_	[64]
Vitamin D3 combined to alginates	Acid and oxidative injury	Induces apoptosis	Activates autophagic and survival pathways	[65]
Chloroquine	Indomethacin	Decreases apoptotic and autophagic cell death	Promotes autophagy	[66]
Chloroquine	Indomethacin	Decreases apoptotic and autophagic cell death	Promotes the expression of Smad7	[67]
LDOP-1	Ethanol	Induces gastric mucosal injury	Promotes autophagy by the AMPK/mTOR signaling pathway	[68]
*AS-IV*	MNNG	Protects the gastric mucosal injury and decreases the occurrence of gastric cancer	Regulates the p53 expression to activate the Ambra1/Beclin1 complex	[69]

## Abbreviations

*H. pylori*: *Helicobacter pylori*
VacA: Vacuolating cytotoxin
CagA: Cytotoxin-associated gene A
LRP1: Low-density lipoprotein receptor-related protein-1
UPR: Unfolded protein response
TRPML1: Transient receptor potential membrane channel mucolipin 1
CAPZA1: Capping actin proteinof muscle Z-line alpha subunit 1
LAMP1: Lysosomal associated membrane protein 1
STAT3: The signal transducer and activator of transcription 3
OTA: Ochratoxin A
NSAIDs: Nonsteroidal anti-inflammatory drugs
EFL1: Euphorbia factor L1
AST: Astaxanthin
PDTC: Pyrrolidine dithiocarbamate
CQ: Chloroquine
LDOP-1: Polysaccharides from *Dendrobium Officinale* Kimura and Migo Leaves
AS-IV: Astragaloside IV
GPL: Gastric precancerous lesions
MNNG: N-Methyl-N′-nitro-N-nitrosoguanidine.
